# Protein Design Using Physics Informed Neural Networks

**DOI:** 10.3390/biom13030457

**Published:** 2023-03-01

**Authors:** Sara Ibrahim Omar, Chen Keasar, Ariel J. Ben-Sasson, Eldad Haber

**Affiliations:** 1 Proteic Bioscience Inc., Vancouver, BC V7T 1C0, Canada; 2 Department of Computer Science, Ben Gurion University of the Negev, Be’er Sheva 84105, Israel; 3 Independent Researcher, Haifa 3436301, Israel; 4 Department of Earth Ocean and Atmospheric Sciences, University of British Columbia, Vancouver, BC V6T 1Z4, Canada

**Keywords:** protein design, physics-informed neural networks, binary optimization

## Abstract

The inverse protein folding problem, also known as protein sequence design, seeks to predict an amino acid sequence that folds into a specific structure and performs a specific function. Recent advancements in machine learning techniques have been successful in generating functional sequences, outperforming previous energy function-based methods. However, these machine learning methods are limited in their interoperability and robustness, especially when designing proteins that must function under non-ambient conditions, such as high temperature, extreme pH, or in various ionic solvents. To address this issue, we propose a new Physics-Informed Neural Networks (PINNs)-based protein sequence design approach. Our approach combines all-atom molecular dynamics simulations, a PINNs MD surrogate model, and a relaxation of binary programming to solve the protein design task while optimizing both energy and the structural stability of proteins. We demonstrate the effectiveness of our design framework in designing proteins that can function under non-ambient conditions.

## 1. Introduction

Protein sequence design is the process of generating a sequence of amino acids that folds into a desired shape to perform a certain function. To achieve this, several approaches have been developed which can be divided into traditional-physics-based e.g., [1,2], and Machine Learning (ML)-based techniques e.g., [3,4,5].

Traditional physics-based methods led to the initial steps in protein design and have contributed significantly to our understanding of protein structure and function. Yet, these methods suffer from several limitations, including model complexity, sensitivity to model accuracy, computational cost, and oftentimes a lack of flexibility. Traditional algorithms often presume a fixed or almost fixed backbone. Such an assumption consequently means that the protein structure is weakly sequence dependent; thus, changing the sequence does not significantly change the structure [2,6,7,8]. This approximation can work only for small changes in the original sequence, or when changes in the original sequence do not break or create any significant new interactions. Indeed, if we arbitrarily change every residue in the original sequence, the structure is almost surely bound to change. Therefore, while this approach can lead to elegant optimization algorithms, it lacks robustness as the number of mutations in the sequence increases. Furthermore, such an approach necessitates a reasonable initial guess, namely a protein that already closely folds into the desired shape, making the use of this approach for *de novo* design difficult.

Machine Learning methods have proven to be effective tools for predicting protein structure and function, as well as designing new proteins [4,9,10,11], yet they avoid energy calculations all-together. Since ML methods do not use energy calculations and since they often lack interpretability and have limited explanatory power, there is no guarantee that the generated structure-sequence model optimizes some target function (e.g., stability), even approximately. Furthermore, even if a sequence does fold into a desired structure it may be unstable or suboptimal.

Physics-based and deep learning-based methods rely on empirical energy functions and machine learning models, respectively. Both are parameterized by the known protein structures, regardless of the optimal conditions for their folding and operations. For most of them, however, these conditions are ambient. Thus, interpolating these functions and models to specific domains of non-ambient conditions (e.g., high temperature) is speculative, and likely wrong.

In this work, we tackle the problem of protein design from a different perspective. Rather than using the commonly used energy functions on a static structure, we use molecular dynamics (MD) simulations to compute the protein energy landscape. In this way, we both avoid the fixed backbone assumption and infer an energy landscape that is MD-derived for a target under specific environmental conditions. Thus, this method should allow us to design proteins that approximately fold into a target structure, optimize their properties even at non-ambient conditions, and account for their flexibility, under the assumption that molecular dynamics simulations faithfully describe protein interactions (see [12,13,14] and reference within).

A similar idea was used in [12]; however, its usability is prohibitively restricted by the computational complexity. Since the energy, structure and stability of the protein are determined by MD, computing them a number of times might not be realistic. We, therefore, extend previous work and combine MD with a deep learning algorithm that approximates the quantities obtained from MD. Replacing MD with ML is a recent practice (see e.g., [15,16] and references within). Unlike many recent papers that aim to replace MD with deep learning to approximate the entire MD trajectory, our model is designed to approximate only the average energy and deviation from the target design, requiring only a small amount of computational effort. Such an approximation is sometimes referred to as a Physics-Informed Neural Network (PINN) or surrogate model (see [15] and references within). Using PINNs as surrogate models is an inexpensive approximation that replaces complex simulations and is now commonly carried out in many fields (see, e.g., [17,18,19,20]), ranging from flow to chemical engineering and hardware testing. We, therefore, find it appropriate to extend this methodology to protein design.

**Figure 1:**
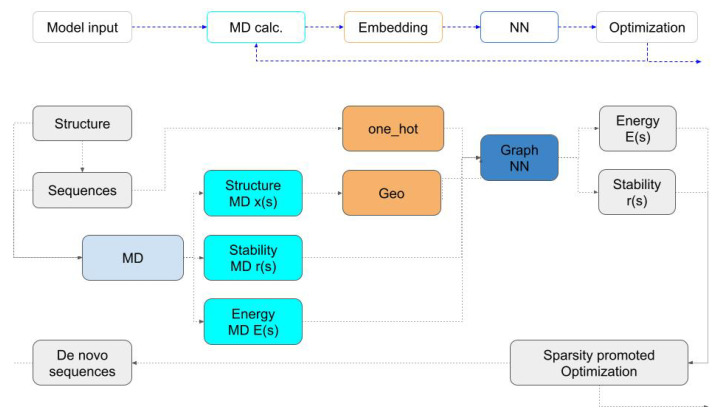
Design workflow. Input model structure and sequences are processed by MD simulations to generate time trajectories of structure, stability, and energy. All metrics are used to establish PINNs, followed by a sparsity-promoted optimization algorithm. The output is either used to retrain the model or outputs the suggested optimized sequences.

We set out to construct PINNs which, given a sequence and MD data, predicts the protein energy, metrics that measure the deviation of the structure from the initial design as well as stability. Once PINNs learn a certain model, they can replace MD and be used to efficiently solve the protein design task as an optimization problem (see algorithm description in Figure 1).

To solve the optimization problem that is associated with the design, we use binary programming where the energy calculations are conducted inexpensively. We generate new, low-energy sequences as we solve the optimization problem. These sequences are simulated by MD and their energy and structure data are input into the networks to be re-trained. The algorithm exploits the neural network surrogate to MD while exploring the space by using random perturbations.

Note that a fundamental aspect of this work is that we aim to generate predictions that are as good as MD predictions. Clearly, if the simulated system is too far from equilibrium or the computed energy is incorrect then our model will fail to reproduce experimental results. However, the calculation of energies based on MD trajectories has been thoroughly studied and therefore can be considered a reliable alternative compared to many ad hoc calculations [12,21,22,23,24].

The rest of this paper is structured as follows. In Section 2, we mathematically define the protein design problem. In Section 3, we describe the surrogate model used to approximate the energy. In Section 4, we describe the optimization procedure used to design new proteins. Section 5 describes a case study and in Section 6 we summarize the paper.

## 2. Mathematical Definition of the Protein Design Problem

In this section, we discuss the mathematical framework for the solution of the protein design problem. While this framework bears some resemblance to earlier studies in this field, there are some substantial differences that make our formulation unique.

Similar to other classical work, the driving force behind our protein design algorithm is the energy of the system. Therefore, a key requirement is to be able to compute the energy for every given sequence. Common codes assume fixed coordinates and compute the energy while others use heuristics to do so. Here, we attempt, for the first time, to introduce protein dynamics in solution under the desired environmental conditions while avoiding common assumptions that could lead to unrealistic designs.

Assume that we have a protein or a number of proteins that interact through some interface. The interface may be intra-chain, or between different proteins in a complex. We represent the system with a sequence, s, and the coordinates of its full atom model, x.

Assume that we have a target structure $x_{\mathrm{tar}}$, to which we aim the protein to fold into. This structure may be fully known, or alternatively, only a portion of the region involved in the interaction might be defined (protein in painting [25,26]). The latter may be the case when considering protein–protein interactions where only the interaction site is known. Our goal is to find sequences $\hat{s}$ with low energies and low structural deviations from $x_{\mathrm{tar}}$.

There are a number of challenges when considering the binding energy and structural stability of a complex. First, there is no unique value to the binding energy. Proteins are flexible and may even change conformation. Second, taking into account the energy alone may not be sufficient. A low energy protein may be structurally unstable/disordered if its energy landscape has several local minima or is very flat, allowing for a high level of coordinate perturbation. We thus turn to a different approach that takes these limitations into consideration. Let us assume that we have a sequence s and its all-atom model. In order to compute the energy of this sequence, we simulate the protein. MD trajectories can be analyzed to obtain the energy of the protein and extract its coordinates for every timestep of the simulation. Since proteins in solution are in constant motion and fluctuate around a minimum at equilibrium, we define the following quantities

(1a) $$\begin{array}{ccc} r(s) & = & \frac{1}{T}\int_{0}^{T}{∥x\left(t\right)-x_{\mathrm{tar}}∥}^{2}\;dt \end{array}$$

(1b) $$\begin{array}{ccc} E^{*}\left(s\right) & = & \frac{1}{T}\int_{0}^{T}E\left(x\left(t\right),s\right)\;dt \end{array}$$

Note that these quantities depend only on s and $x_{\mathrm{tar}}$ as the coordinates x(t) are integrated out. The first quantity measures the average deviation of the coordinates from the desired fold, the Root Mean Square Deviation (RMSD), from the target. This deviation is small in stable proteins with a fold, which is similar to the desired design. However, for unstable proteins or ones that undergo large conformational changes, this quantity may be rather high. We refer to the RMSD as stability. The second component, $E^{*}\left(s\right)$, is the average energy of the structure.

Let us now consider the design of a new protein. In this case, we want to design a protein with a particular structure of interest $x_{\mathrm{tar}}$. Our goal is to find an optimal sequence $\hat{s}$ with two characteristics. First, we require that the energy of the protein $E^{*}\left(\hat{s}\right)$ is low. Second, we require that its structure is stable around the required fold, i.e., $r(\hat{s})$ is small. This strategy permits coordinate motion (no fixed backbone assumption) and also addresses the problem of structural instability in the designed structure. These requirements lead to the following optimization problem

(2a) $$\begin{array}{ccc} \min_{s}\;\; & & E^{*}\left(s\right)\;\;\;\;\;\mathrm{energy}\;\mathrm{minimization} \end{array}$$

(2b) $$\begin{array}{ccc} s.t.\;\; & & r\left(s\right)\leq \delta_{\mathrm{stab}}\;\;\mathrm{structural}\;\mathrm{stability} \end{array}$$

where δ is some acceptable deviation from the target structure.

The optimization problem (2a) and (2b) is challenging to solve. The difficulty stems from two main issues. First, the energy and stability are computed by expensive simulations. While this looks initially difficult, deep learning approximations have been developed to replace expensive MD at a fraction of the cost in recent years. We thus use MD simulations to train a graph neural network that can approximate those simulations, yielding the desired quantities for the solution of the problem. Note that our network is different from networks that recover the complete trajectory. Our demands are substantially modest as we aim to approximate only the average energy of the protein and its coordinate deviation from the target design.

A major challenge in such an optimization task is the discrete nature of the sequence s, which can be solved as a binary programming problem. To address this, we utilize a sparsity-promoting optimization method that employs convex relaxation to replace the binary optimization problem. This approach has proven to be effective for several binary optimization problems [27,28,29]. Although the method may require intensive sampling, it is highly efficient when the objective function evaluation is inexpensive. As a result, our approach of combining this method with surrogate deep learning models can be considered an effective solution for the protein design problem.

## 3. Deriving a Surrogate Neural Network

Since solving the optimization problem requires the MD simulation of every given sequence-structure set, and since each MD simulation is computationally intensive, we opt for a so-called surrogate deep learning model to replace the MD simulation. The goal of the surrogate is to predict the binding energy $E^{*}\left(s\right)$, and the structural stability ∥r(s)∥ for a given protein. These quantities can then be used directly in the optimization problem (2a) and (2b), saving on the time costs of MD simulations and significantly reducing the cost of the optimization problem.

Protein folding is influenced by both local and global interactions between residues, making it a system that can be described well using a graph. We experimented with graph neural networks (GNNs) [9] and selected the one proposed in [30] as it has an interpretation of energy.

The input to the network is the sequence (one hot encoded) and the coordinates of the alpha carbon ($C_{\alpha }$) atom of each residue. Given the coordinates, we compute the distance matrix (that is, the distance between every pair of $C_{\alpha }$ atoms) and use the distance map as a soft adjacency matrix. From the coordinates, we further derive a number of invariant features. To this end, we use the Frenet framework [31] that uniquely defines any curve in 3D space up to rotation and translation. We compute the curvature of each protein, κ(x), and its torsion τ(x). We use these features, together with one hot embedding, as inputs to our network.

The network can be written as

(3) $$\begin{array}{ccc} Y_{0} & = & K_{0}X \end{array}$$

(4) $$\begin{array}{ccc} Y_{j+1} & = & Y_{j}+\sigma \left(K_{j}Y_{j}L\right)\;j=1,\ldots ,N\\ X_{\mathrm{out}} & = & K_{\mathrm{out}}Y_{N} \end{array}$$

Here, X, which represents the sequence, is a matrix of k×n, where *k* is the number of possible amino acids (typically 20), *n* is the length of the protein, L is the n×n graph Laplacian matrix, the weights $K_{j}$ are s×s and $K_{\mathrm{out}}$ is a 2×s matrix. We choose σ(·) to be the leaky Relu function.

The output of the network, $X_{\mathrm{out}}$, contains two channels. The first is the binding energy of each residue and the second is the RMSD of each residue. When we train the network, we minimize the difference between $X_{\mathrm{out}}$ to the per node energy and structural deviation obtained by the MD simulation.

Thus, if the network is trained successfully, it can approximate both the binding energy and the structural stability of a given sequence; it can therefore be used for protein design without the computational cost of MD simulations.

## 4. Binary Programming Using Group Sparsity Relaxation

The core of the protein design problem requires solving an optimization problem based on a specific energy. The sequence s is converted into a s×n “one hot” matrix S, where each column represents a residue and each entry is binary. Techniques for solving binary optimization include relaxation and random searches [27,28,29]. Here, we propose a modification of the concept of group sparsity to approximately solve the optimization problem, as it has been successful in relaxing non-convex problems in the past (see [32,33,34] and reference within).

To this end, rather than solving for a binary matrix S that has a single non-zero entry at each column we define the convex set, S and require the following three properties for every matrix S∈S:

(5) $$\begin{array}{ccc} & & 0\leq S_{ij}\leq 1 \end{array}$$

(6) $$\begin{array}{ccc} & & \sum_{i}S_{ij}=1 \end{array}$$

(7) $$\begin{array}{ccc} & & {∥S∥}_{1,2}^{2}=\sum_{j}{\left(\sum_{i}S_{ij}\right)}^{2}\leq n \end{array}$$

The first two properties simply imply that each element in the matrix is between 0 and 1 and that the sum of each column of the matrix is also 1. The last term in the set is the so called $\ell_{1,2}$ norm and its role is to promote sparsity in each column of the matrix. Since the matrix S is sparse, holding exactly *n* non-zero entries, group sparsity yields a structure of S that is similar to the one obtained from binary programming.

The set S is convex; therefore, the projection is well defined (see [34] for details). To this end, we define the projection operator as

(8) $$\begin{array}{c} P_{S}\left(S^{*}\right)=\arg\min_{S\in S}\frac{1}{2}{∥S-S^{*}∥}^{2} \end{array}$$

The projection takes a matrix $S^{*}$ that is outside the convex set S and solves for a matrix S that belongs to the set S. As shown in [34], this can be achieved using simple thresholding. Given the projection $P_{S}$, we can now use the gradient projection algorithm [35] for the design problem. The algorithm takes a steepest descent step to minimize the energy followed by a projection to the set S (see Algorithm 1).

Convergence occurs when S is a fixed point of the iteration. However, we use another important criterion to terminate the algorithm and exit. Recall that the energy we use in this algorithm is not the true energy but rather a surrogate function. When training the network, we keep track of our validation accuracy and the number of mutations in S. The network is trained until the number of mutations exceeds a preassigned number. Here, we allow up to 10 mutations in the protein and exit the algorithm when it exceeds this number. While there is no proof that such an algorithm converges to an exact solution, we have found that after a small number of iterations the algorithm does not yield significant improvement in the energy and yields acceptable solutions. These findings are consistent with other applications [36] where similar algorithms have been used. Notably, each iteration of the algorithm requires an evaluation of the energy function, which is prohibitively expensive if MD is used, but feasible with the surrogate model. Thus, practical design is obtained in linear time with respect to the length of the protein.
**Algorithm 1** Group Sparsity Design**Require:**S,X, niter, $\delta_{\mathrm{stab}}$n=length(X)E←∞set $S\leftarrow P_{S}\left(S\right)$**for**i=1…niter**do**    Using the network compute E(S,X) and $\nabla_{S}E$    $S\leftarrow P_{S}\left(S-\mu \nabla_{S}E\right)$    Check for convergence or exit conditions.**end for**return S

## 5. Example: Designing Cardiac Troponin Binders

Cardiac troponin I (cTpnI) is a protein found in heart muscles [37]. Upon damage to cardiac cells such as in acute myocardial infarction (AMI) (commonly known as heart attack), cTpnI is released in the blood [38]. Hence, the detection of cTpnI makes it an excellent biomarker for the diagnosis of AMI [39,40,41]. In this section, we describe cTpnI binders design using our PINNS approach.

**Figure 2:**
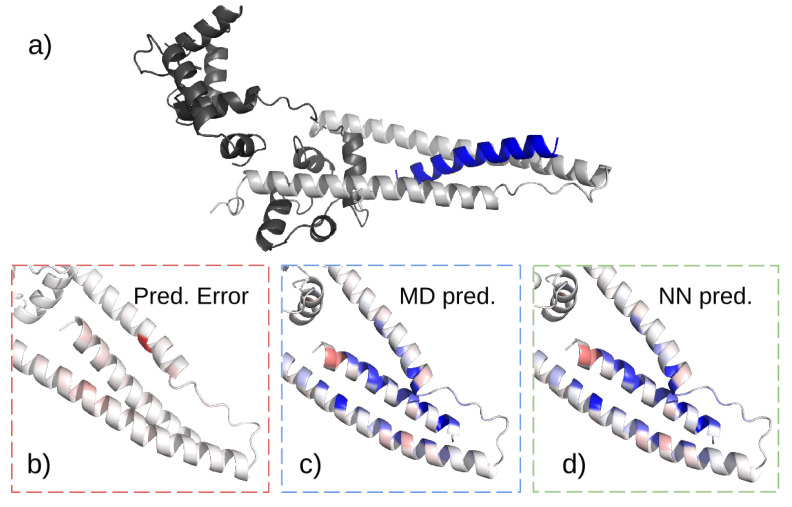
Structure of the Troponin complex. (**a**) Ribbon representation of a modified Troponin complex. Using a fragment of Troponin T (blue) as a template, the design of a binder for Troponin I (light gray)-Troponin C (dark gray) complex can be optimized. (**b**) Mean normalized energy prediction error (white_red spectrum, red indicates large errors) between MD prediction (panel (**c**)) and NN prediction (panel (**d**)), (both panels are blue_white_red spectrum, blue indicates negative energy values). Note that the overall error is low, as indicated from the minor color difference representing the energy predictions even where the difference (normalized) is the most significant. This means the model can be used for optimization.

In cardiac cells, cTpnI forms a complex with the following two regulatory proteins: troponin C (TpnC) and troponin T (TpnT) [38]. The crystal structure of the ternary complex is available (Figure 2a) [42]. In blood, however, the biomarker can be found in a complex with either TpnC only or both TpnC and TpnT. Our goal is to design peptides that can specifically bind to cTpnI complexed with TpnC, herein referred to as the target complex. Such a binder can be used for the detection of cTpnI to test for AMI.

### 5.1. Choice of the Template Peptide

This study is based on the experimentally-resolved structure of the cTpnI-TpnC-TpnT complex, PDB ID: 1J1D [42]. Our first goal was to choose the fragment of TpnT that would be the starting template for the designed binder. An ideal binder would have high affinity and selectivity to cTpnI. Therefore, we aim to find the highest affinity fragment of TpnT to cTpnI-TpnC. Specifically, we ran MD simulations of the troponin complex. The average energy contribution of each residue of TpnT to the total binding energy was calculated to decide the highest affinity fragment to be used as a design template.

To prepare the complex for MD simulations, we first protonated the 1J1D structure using PDB2PQR [43,44] with the Amberff14SB forcefield [45]. This complex was neutralized and solvated using the tleap program of Ambertools21 [46], in TIP3P 0.15 M NaCl water box. The solvated system was parameterized using the Amberff14SB forcefield [45] and simulated using the NAMD simulations package [47]. First, water molecules and ions were minimized for 1000 steps while the protein complex was restrained. This was followed by 1000 step minimization of the unrestrained system. Next, the backbone-restrained system was gradually heated from 0 to 300 K. The restraints were then gradually decreased until they were completely removed. The non-restrained system was finally simulated for 500 ns. The MMPBSA.py program [48] was used to calculate the Molecular Mechanics Generalized Born Surface Area (MMGBSA) binding energy (BE) of TpnT to the target complex. Generally, the change in Gibbs free energy ($▵G_{bind}$) can be approximated as follows:

(9) $$▵G_{bind}=▵H-T▵S\approx ▵E_{MM}+▵G_{sol}-T▵S$$

in which ▵H is the change in enthalpy, *T* is the temperature, ▵S is the change in entropy, $▵E_{MM}$ is the molecular mechanics energy in the gas phase and $▵G_{sol}$ is the solvation free energy. BE is computed using MMPBSA.py and is calculated as follows:

(10) $$\begin{array}{c} BE=▵E_{MM}+▵G_{sol} \end{array}$$

(11) $$\begin{array}{c} ▵E_{MM}=▵E_{inter}+▵E_{elec}+▵E_{vdW} \end{array}$$

BE is a sum of $E_{inter}$, which is the internal energy of the molecule bond distances, angles and dihedrals, as well as $▵E_{elec}$ and $▵E_{vdW}$, which are the electrostatic and van der Waals energies. Note that the entropic change is not accounted for in this calculation since such a calculation is computationally demanding and inaccurate.

BE was decomposed to each residue of TpnT and it was decided that residues 104–130 would constitute the starting template for our peptide design, herein referred to as the TpnT-template.

### 5.2. Generating Training Data

While it is possible to randomly sample peptide structures and compute their trajectories, it is better to sample structures that are considered feasible, that is, those structures that do not diverge when running molecular dynamics. As explained in the introduction, there are many software packages that can provide an initial design using either empirical energy or machine learning. Here, we use the popular Rosetta package and its protein complex generation process [7].

The proteins cTpnI and TpnC complexed with residues 104–130 of TpnT were first repacked and minimized based on the Beta_nov16 scoring function [49]. This was followed by Monte–Carlo based mutagenesis of the design peptide; in this step, the electrostatic contribution of the Beta_nov16 scoring function was up-weighted by a factor of 1.5. This modification to the scoring function biases mutagenesis towards residues that would form more hydrogen bonds, as this would increase the designed peptide specificity. The mutant fragment was then redocked to the target complex and minimized before the final complex structure was output. A total of 15,000 design attempts were made using Rosetta. Only about 2600 designs passed the following initial screening criteria: general design score of −190 Rosetta Energy Units (REUs), a binding energy of −50 REU, a shape complementarity score of 0.75 and no more than 3 buried unsatisfied hydrogen bonds. These 2600 structures were further filtered using MD simulations. Each designed peptide complexed with cTpnI-TpnC was prepared for MD simulations, as described in Section 5.1. Each complex was neutralized and solvated in a TIP3P 0.15 M NaCl water box. The backbone-restrained system was minimized for 1000 steps followed by another 1000 minimization steps without restraints. The backbone-restrained system was then heated from 0 to 300 K; then, the restraints were incrementally removed at 300 K. The unrestrained complex was simulated for 5 ns. Data from 5 ns simulations were extracted for the training of our algorithm.

Three pieces of data were used to train our algorithm.

- Complex coordinates were extracted from the 5 ns simulations at regular intervals for a total of 50 coordinate sets per designed peptide. These were used to compute the center of mass coordinates, $x^{*}\left(s\right)$, as well as standard coordinate deviation during simulations, r(s), of the trajectory.
- The average MMGBSA binding energies of the designed peptides to the target complexes, representing $E^{*}\left(s\right)$, were computed using MMPBSA.py [48].
- The contribution of each individual residue to the average MMGBSA binding energy for each complex was also calculated.

As part of trajectory analysis, we also computed the MMGBSA binding energies for the 2600 complexes over the 5 ns simulation time. While more accurate methods for calculating binding energies exist, such as thermodynamic integration [50,51] and free energy perturbation [52], these methods are computationally demanding and would be impractical for calculating the binding energy of thousands of complexes. Furthermore, previous studies have shown that the Molecular Mechanics Poisson Boltzmann Surface Area (MMPBSA) method for calculating the binding energy was comparable to MMGBSA, with the latter being less computationally expensive [53,54].

**Figure 3:**
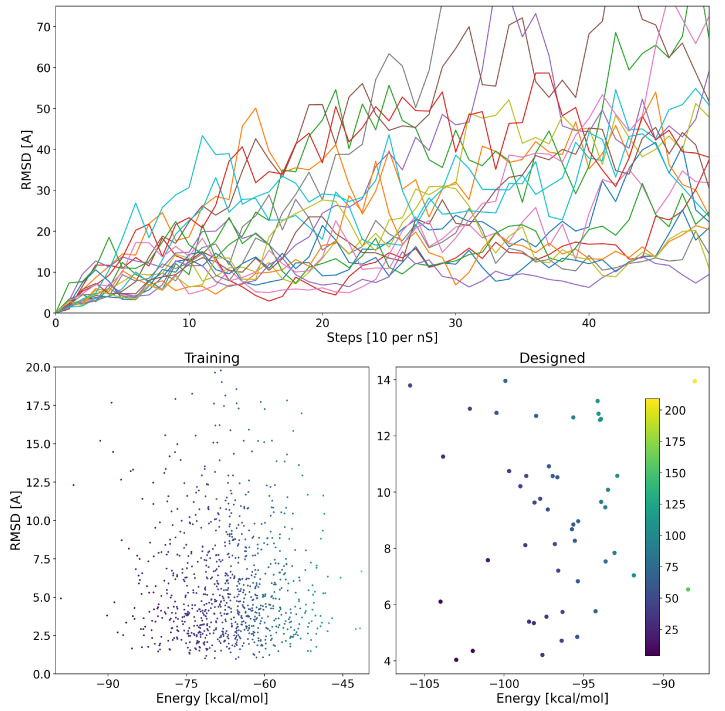
**Top** panel: The trajectories of RMSD of 10 random complexes over 5ns. It is evident that some designs are more stable and remain within their original conformation from design (low RMSD), whereas others deviate greatly from their original design coordinates (high RMSD). **Bottom** panel: The binding energy of different complexes and their corresponding coordinate RMSD. The best designs (low energy and stable) are plotted in blue while less reliable ones become more yellow.

We also calculated the RMSD of the design atomic coordinates over the simulation time. In Figure 3, we plot the RMSD as a function of time for 10 random designs. The RMSD of some structures continued increasing, which implying that they were unstable. For other structures, however, we observe an initial structural drift followed by a plateau in RMSD values. It is important to note that 5 ns is a short time to deem whether RMSD plateauing determines structural stability. However, such short simulation times provide a means of filtering initial designs within reasonable computational costs. RMSD values were condensed to the vector r with Equation (1a), and were predicted using the network.

An ideal design would have a low binding energy and would not considerably deviate from the original design coordinates. We plot the MMGBSA binding energy of 950 structures verses their RMSD in Figure 3. It can be observed that there is no correlation between a designed peptide average MMGBSA binding energy and its structural stability during the simulation. Hence, it is important to compute and evaluate both attributes in the complex to assess design success.

### 5.3. Training the Model

Given the training data, we trained the graph neural network described in Section 3. In the first step, we take the energy and the stability data, subtract the mean and divide by the standard deviation. This is a common practice in most training procedures [55]. The network has a total of roughly 1.3 M parameters, and we use 5 layers of the graph network. The opening layer, $K_{o}$, increases the size of the network from 23 channels (20 for the one hot sequence and 3 for the geometrical features) to 512 channels. Using this architecture, we manage to reduce the error of the validation data set to $9.8\times 10^{-3}$. The maximal error in training is plotted in Figure 2.

### 5.4. Using the Deep Learning Model for Protein Design

**Figure 4:**
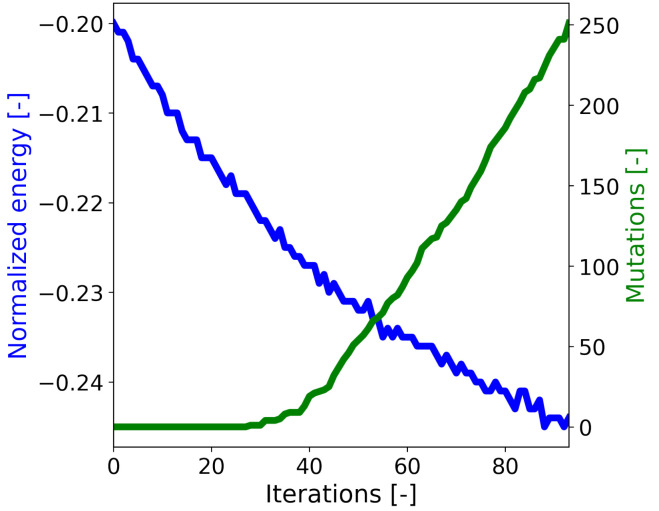
Convergence of the optimization algorithm and the number of mutations observed by using Algorithm 1. Note that the presented energy is the normalized one (de-biased). The code is terminated when 5 mutations are proposed on average, leading the overall mutations over 50 designs to amount to 250.

Given the initial 950 sequences, we chose the best 50 sequences and used the optimization Algorithm 1. We used a total of 5 mutations in each protein before the optimization algorithm returned. A graph of the energy as a function of iterations as well as the number of total mutations (the number of mutations in all designs) is presented in Figure 4.

**Figure 5:**
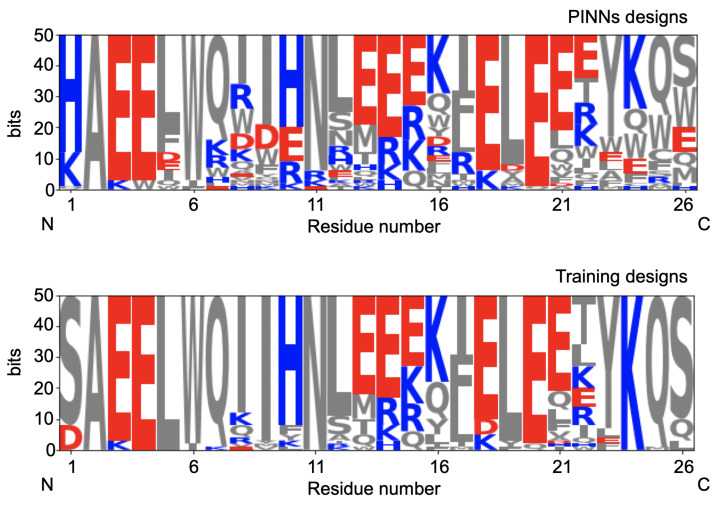
Design sequence logos of the 950 designs from the training set (**bottom**) and the PINNs-generated designs (**top**). Blue, red and grey colors indicate that the residues are positively charged, negatively charges or uncharged, respectively. It is evident that the PINNs designs are more diverse compared to the initial designs from the training set. Since the problem is highly non-convex, this allows us to generate many different sequences with different starting points.

The initial 50 sequences and the designs obtained by using the algorithm are plotted in Figure 5. We observe a much larger variability of designs compared with the initial designs.

The newly designed proteins are then used as validation data. The energy of the designed proteins as well as their structural stability is plotted in Figure 3. We see an overall agreement between the predicted and observed energies and stability.

## 6. Summary

In this work, we proposed a new framework that combines MD, deep learning, and binary programming optimization that leverage the strengths of each to solve the protein design task. Our goal is to use the most accurate energy model possible in order to achieve a stable design. To this end, we use MD to account for the protein environment and flexibility, since proteins are in constant motion. The MD trajectories are used for the calculation of an average binding energy. To assess the structural stability of the protein, we also record the deviation of the designed protein from the desired design. The average binding energy and RMSD of each sequence are then approximated using a graph neural network. The goal of the network is to recover the average binding energyand the average deviation of the design, which is more achievable than recovering the whole simulation. This approach is commonly referred to as PINNS and has gained popularity in many science and engineering problems. We train a graph neural network on data that are generated using MD. The resulting network is then used for solving the protein sequence design problem. For optimization, we use a convex relaxation of the problem with a sparsity-promoting norm. This yields sparse designs that are used to probe the energy surface. The code was tested on a true design of a binder to troponin. We have shown that we are able to find designs that indeed have a low binding energy (as computed by MD) given the trained data. Our designs reduce the energy by an average of 20% while keeping the protein stable. Our results yield a more diverse set of sequences compared with the initial designs, which will facilitate the laboratory testing of many more variants compared with the initial variants.

Our method heavily relies on MD, which accounts for protein flexibility. It is worth noting that simulation conditions can be set to facilitate the evaluation of designed proteins at different temperatures. Furthermore, different pH values may be considered by adjusting residue protonation. We assumed in this procedure that if the structure for a given sequence stably binds to the target protein with high affinity then it will manifest this prediction in the lab. On the other hand, if the simulations were not reliable our design would lead to unreliable binder sequence predictions. Clearly, any final design needs to be validated in the laboratory. It is important to note that 5 ns simulations are too short to assess the structural stability of peptide–protein complexes. Longer simulation times could be employed to address this issue although this would be computationally expensive. Alternatively, longer simulations could be obtained for filtered designs that have an initially low RMSD. Validating MD results against laboratory experiments is beyond the scope of this paper. However, recent computational techniques are proposed to obtain an even higher accuracy for MD when solving the full Schrödinger system. Our approach, which can be used with any energy function, is designed with the aim of reducing the need for laboratory experiments by improving the accuracy of protein design simulations.

## Data Availability

Data is available on a Github repository.
